# Parenting under pressure: a cross-sectional questionnaire study of psychological distress, parenting concerns, self-efficacy, and emotion regulation in parents with cancer

**DOI:** 10.2340/1651-226X.2024.40404

**Published:** 2024-06-20

**Authors:** Maria Romare Strandh, Pia Enebrink, Karin Stålberg, Renita Sörensdotter, Lisa Ljungman, Anna Wikman

**Affiliations:** a Department of Women’s and Children’s Health, Uppsala University, Uppsala, Sweden; b Centre for Women’s Mental Health during the Reproductive Lifespan (WOMHER), Uppsala University, Uppsala, Sweden; c Department of Clinical Neuroscience, Karolinska Institutet, Stockholm, Sweden; d Centre for Gender Research, Uppsala University, Uppsala, Sweden

**Keywords:** Neoplasms, parents, parenting concerns, psychological distress, depression, anxiety, stress

## Abstract

**Background and purpose:**

As many as one in four adults with cancer have children under 18 years.

Balancing parenting and cancer is challenging and can be a source of psychological distress. This study aimed to examine psychological distress in parents with cancer and its associations with parenting concerns, self-efficacy, and emotion regulation.

**Materials and methods:**

This was a cross-sectional questionnaire study of 406 parents (aged 25–60 years) diagnosed with cancer within the last 5 years, with at least one dependent child (≤ 18 years). Parents completed questionnaires on psychological distress (DASS-21), parenting concerns (PCQ), self-efficacy (GSE), emotion regulation (ERQ), mental and physical health, and sociodemographics. Data were analysed using multiple logistic regressions on depression (yes/no), anxiety (yes/no), and stress (yes/no).

**Results:**

Higher parenting concerns were associated with greater odds of depression (OR = 2.33, 95% CI: 1.64–3.31), anxiety (OR = 2.30, 95% CI: 1.64–3.20), and stress (OR = 3.21, 95% CI: 2.20–4.69) when adjusting for health and sociodemographic factors. Poorer self-efficacy was associated with increased odds of anxiety (OR = 0.94, 95% CI: 0.89–0.99, p < 0.05), whereas lower use of cognitive reappraisal and higher use of expressive suppression increased the odds of depression (OR = 0.76, 95% CI: 0.59–0.98 | OR = 1.46, 95% CI: 1.18–1.80).

**Interpretation:**

The findings highlight the complexity of parental well-being in relation to parenthood and cancer, stressing the need for interventions that address relevant psychological factors to improve overall mental health in this population.

## Graphical abstract

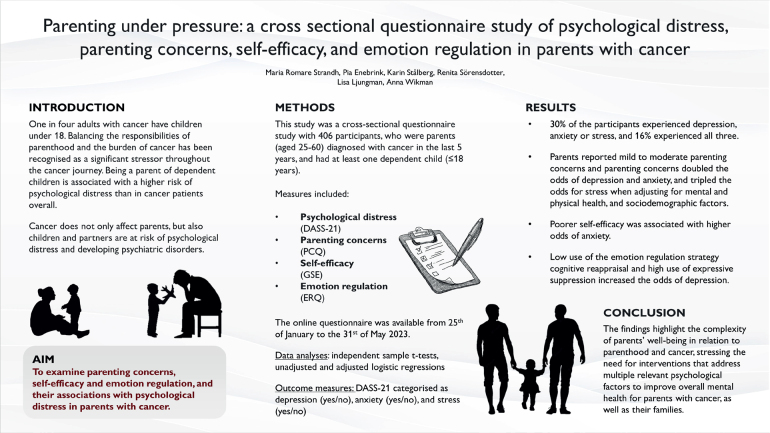

## Introduction

Cancer represents an increasing global challenge, with 19.3 million new cases diagnosed worldwide every year [1]. Receiving a cancer diagnosis is often a shocking life event and 30–60% of adults with cancer experience psychological distress during their cancer journey [2, 3]. Their risk of developing a mental disorder is higher when compared to adults without cancer, with a two to three times greater risk of depression [4].

As many as one in four (24%) of adults diagnosed with cancer have at least one child under the age of 18 years [5]. To be a mother with breast cancer, or a parent with advanced cancer, is associated with an even higher risk of psychological distress than patients with breast cancer or advanced cancer who are not parents [6, 7]. Overall, among parents with cancer, psychological distress is more profound among single parents and when a shorter time has passed since diagnosis [8]. A recent review on the impact of cancer on parents’ mental health reported varied prevalence rates, partly due to heterogeneous study designs, ranging from 4 to 59% for elevated levels of depression, from 7 to 93% for probable depression, and from 11 to 57% for anxiety symptoms, while from 19 to 88% met the criteria for an anxiety disorder [8]. In addition, cancer does not only affect the parents but also their family, where children of parents with cancer are also at risk of psychological distress [5, 9]. The psychological well-being of parents is closely related to the well-being of their children [10], making the parenting aspect of the cancer journey pivotal.

The importance of parenting has been shown in several studies [11]. Among other things, parents have been shown to opt for more aggressive treatments motivated by the desire to survive for their children [12]. Further, around a third of cancer survivors report being somewhat to highly concerned about the emotional and practical impact on their children as a consequence of cancer [13]. Parenting concerns, often studied from the perspective of mothers with cancer [11, 14–17], include feelings of inadequacy in fulfilling the parental role, the potential negative impact of the parents’ illness and possible death on their children, and challenges in communicating with their children about their illness [11, 12, 18, 19]. Parenting concerns are related to higher psychological distress and lower quality of life in parents with cancer [7], as well as lower parenting self-efficacy – which is the confidence and trust parents have in their ability to care for their children in a good way [19]. Low parenting self-efficacy can lead to lower parenting competence [20]. Further, maladaptive emotion regulation has also been associated with psychological distress in parents with cancer [21]; it is a predictor of parenting stress [22] and can be a mediator between resilience and distress [23]. However, children have also been shown to have a positive impact during a parent’s cancer journey [24]. For example, children provide a reason to stick to everyday routines and engage in family activities, which may improve psychological well-being. While having children may be a protective factor, balancing the responsibilities of parenting with the burden of cancer is challenging, and has been recognised as an immense stressor throughout the cancer journey [10, 11].

Although a range of physical, cognitive, and emotional changes during the cancer journey affect psychological distress in parents with cancer [2,18], the particular modifiable factors that need to be targeted in order to reduce their psychological distress in this population are not fully understood [10]. Since parental cancer is a risk factor for psychological distress, both for parents and children, psychological mechanisms such as self-efficacy beliefs, emotion regulation capacity, and importantly parenting concerns that can influence parents’ psychological well-being need to be further investigated in order to develop interventions targeting these. In addition, experiencing higher psychological distress increases the risk of mortality and lower survival time [25], further stressing the importance of addressing psychological distress in parents with cancer. Achieving an understanding of the complexity of the psychological well-being of parents with cancer is crucial for early detection of issues and the development of effective psychological support interventions that can be integrated into cancer care. The aim of this study was, therefore, to examine factors associated with psychological distress in parents with cancer. More specifically, the study explored whether parenting concerns, psychological factors (e.g., self-efficacy and emotion regulation), health factors (e.g., cancer stage and self-rated health), and/or sociodemographic factors (e.g., age, gender, civil status, and number of children) were associated with psychological distress (depression, anxiety, and stress) among parents with cancer.

## Materials and Methods

This was a cross-sectional questionnaire study that was approved by the Swedish Ethical Review Authority (Reference number: 2022-03088-01).

### Participants and procedures

Participants were a convenience sample of parents (aged 25–60 years) diagnosed with any type of cancer within the last 5 years who had at least one child aged 18 years or younger. Time since cancer diagnosis was defined as from the date of primary diagnosis or from the date of the last recurrence. The time frame was chosen to be able to study experience more closely or distantly related to diagnosis and primary treatment in relation to the outcomes. If the parents had received multiple cancer diagnoses, the most current diagnosis was reported.

The questionnaires were selected based on previous work [24, 26], and in collaboration with six research partners (parents with cancer) involved in the project. In addition, the research partners helped with the phrasing of further study-specific self-report questions and piloted the questionnaire to assess relevance, acceptability, and time allocation to complete (20–30 min). The survey tool REDCap was then used to format the questionnaire for use online.

Data collection was carried out between the 25th of January and 31st of May 2023. Participants were recruited via advertisements distributed by patient organisations, on social media, and advertising posters were sent to the six Regional Cancer Centres in Sweden who were asked to distribute these to the oncology clinics in their region for display at their clinics. The survey was available on a dedicated project website where more information about the study was available, as well as the contact details of the research team. Participants received a gift voucher of 200 SEK after they completed the questionnaire.

### Measures

#### Outcome variables

*Psychological distress:* Psychological distress was measured using the Depression, Anxiety, and Stress Scale-21 (DASS-21) [27]. DASS-21 consists of 21 items scored on a 4-point Likert scale from 0 (*did not apply to me at all*) to 3 (*applied to me very much or most of the time*), designed to measure the severity of symptoms related to depression, anxiety, and stress, with higher scores indicating greater symptom severity. The three subscales are scored separately (depression [D], anxiety [A] and stress [S]), and have a total score ranging from 0 to 42 (scores are multiplied by 2 as this is a short version of DASS). Each subscale has separate cut-off scores defining symptoms as normal (D: 0–9, A: 0–7, S: 0–14), mild (D: 10–13, A: 8–9, S: 15–18), moderate (D: 14–20, A: 10–14, S: 19–25), severe (D: 21–27, A: 15–19, S: 26–33), or extremely severe (D: > 28, A: > 20, S: > 34). For the purpose of this study, moderate symptoms and above were defined as the cut-off for depression, anxiety, and stress respectively, since these were considered clinically relevant levels of psychological distress.

#### Study variables

*Parenting concerns: Parenting concerns in parents with cancer were assessed using the* Parenting Concerns Questionnaire (PCQ) [28], which was developed for patients with cancer and consists of 15 items on three subscales: *practical impact of illness on the child/ren*, *emotional impact of illness on the child/ren*, and *concerns about the co-parent*. Participants responded on a 5-point Likert scale from 1 (*not at all concerned*) to 5 (*extremely concerned*), where a higher score indicates greater parenting concerns. Total scores can range between 1 and 5. In this sample, the internal consistencies for all subscales were acceptable or very good (practical impact: α = 0.85; emotional impact: α = 0.85; co-parent: α = 0.79; total score: α = 0.89) [29].

*Self-efficacy*: Self-efficacy was measured using the General Self-Efficacy Scale (GSE) [30]. This scale comprises 10 items assessing an individual’s belief in their ability to cope with challenging situations. Participants responded on a 4-point Likert scale from 1 (*not true at all*) to 4 (*exactly true*), with higher scores indicating higher self-efficacy. The total score can range between 10 and 40.

*Emotion regulation:* Emotion regulation was assessed using the Emotion Regulation Questionnaire (ERQ) [31]. The ERQ has 10 items and measures individual differences in two emotion regulation strategies: *cognitive reappraisal* and *expressive suppression*. Cognitive reappraisal is a strategy to change one’s perspective of a situation that evokes emotions to modify the emotional response, for example interpreting a stressful situation as valuable (e.g., speaking in front of a group of people is a good way to express your opinion, even if it makes you feel anxious). Expressive suppression is a strategy where you modify the emotional expression by concealing your inner emotions (e.g., pretend to be calm when talking in front of other people although you feel anxious). Participants responded on a 7-point Likert scale ranging from 1 (*strongly disagree*) to 7 (*strongly agree*). Total scores can range from 1 to 7 and higher scores indicate a greater use of the emotion regulation strategy.

The questionnaires also included study-specific self-report questions gathering information about *mental health history* (psychological distress before cancer diagnosis), *health factors* (self-rated health, time since diagnosis, and cancer stage), and *sociodemographic factors* (age, gender, education, civil status, number of children, and age of youngest child) that were used as study variables.

### Statistical analysis

Means (*M*) and standard deviations (*SD*) were used to describe numerical variables, and numbers (*n*) and percentages (%) were used for categorical variables. Independent samples *t*-tests were conducted to compare the mean scores on PCQ (total), self-efficacy, and ERQ (cognitive reappraisal and expressive suppression) between those scoring below or above cut-off for symptoms of depression, anxiety or stress, or below or above cut-off on all three DASS-21 subscales. Unadjusted logistic regressions were carried out to examine associations between each study variable separately and the likelihood of scoring above cut-off on depression, anxiety, or stress, or scoring above cut-off on all three dimensions. In adjusted logistic regression analyses, all study variables were included simultaneously in the models to test the association between each study variable in relation to the outcomes, while accounting for the influence of all other study variables in the model. The overall model fit was assessed using the Hosmer–Lemeshow test. The alpha level for all analyses was set at 0.05 with 95% confidence intervals (CI). All analyses were carried out using IBM SPSS Statistics (Version 28).

## Results

### Participant characteristics

The sample consisted of 406 parents with cancer. Participants’ characteristics are shown in Table 1. Participants had a mean age of 45 years (*SD* = 6.4), most were mothers (*n* = 388, 96%), in a partnered relationship (*n* = 336, 83%), and had finished post-secondary education (*n* = 338, 83%). The families had on average 2.2 (*SD* = 1) children and the mean age of the youngest child was 10.6 years (*SD* = 4.9). The majority of parents had breast cancer (*n* = 274, 67%), and 72 parents (18%) reported having incurable cancer. More participants reported psychological distress after receiving a cancer diagnosis compared to before (*n* = 148, 36% before and *n* = 248, 61% after).

**Table 1:** Participant characteristics (*n* = 406).

Sociodemographic characteristics and health status	*n*	%	Mean	SD
**Age, years**			45	6.4
<40	77	19		
40–50	245	60		
>50	84	21		
**Parent with cancer**				
Mother	388	96		
Father	18	4		
**Children**				
Total no. of children	432			
Number of children per family			2.2	1
Age of youngest child			10.57	4.9
**Civil status**				
Single parent	70	17		
In a partnered relationship	336	83		
Relationship length				
<2 years	8	2		
2–10 years	55	14		
>10 years	273	67		
**Education level**				
Secondary education	68	17		
Post-secondary education	338	83		
**Time since cancer diagnosis, years**	1.40	1.4		
<1	146	36		
1–2	163	40		
3+	97	24		
**Cancer diagnosis**				
Breast cancer	274	67		
Central nervous system (CNS) cancer	7	2		
Gynaecological cancer	31	8		
Hematological cancer	19	5		
Skin cancer	16	4		
Lung cancer	5	1		
Upper gastrointestinal (GI) cancer	13	3		
Bowel cancer	27	7		
Urological cancer	6	1		
Other*	8	2		
**Self-reported cancer status**				
Curable				
Yes	334	82		
No	72	18		
Treatment status				
Under treatment	244	60		
Treatment completed	162	40		
Cancer recurrence, yes	53	13		
**Self-rated health**				
Good	228	56		
Poor	178	44		
**Self-reported mental health status**				
Psychological distress before cancer diagnosis				
Yes	148	36		
No	258	64		
Psychological distress after cancer diagnosis				
Yes	248	61		
No	158	39		

* Head and neck cancer, thyroid cancer, sarcoma, and squamous cell cancer.

### Psychological distress, parenting concerns, self-efficacy, and emotion regulation

Levels of psychological distress, parenting concerns, self-efficacy and emotion regulation are presented in Table 2. On the DASS-21 subscales, 140 (35%) parents scored above the cut-off for depression, 127 (31%) for anxiety, and 107 (27%) for stress. Sixty-four (16%) parents scored above cut-off on all three sub-scales, while 19–21% scored above cut-off on two dimensions (D + A, *n* = 86, 21%; D + S, *n*= 81, 20%; A + S, *n* = 77, 19%). Parents reported mild to moderate parenting concerns (total score *M* = 2.31, *SD =* 0.79). Self-efficacy was fairly high (*M* = 28.36, *SD* = 5.09) and parents reported greater use of the emotional regulation strategy cognitive reappraisal (*M* = 4.54, *SD* = 1.10) than expressive suppression (*M* = 3.03, *SD =* 0.98). Table 3 shows parents’ ratings on the PCQ, GSE, and ERQ, comparing those scoring above or below cut-off on the DASS-21. Parents scoring above cut-off reported greater parenting concerns, lower self-efficacy, used less cognitive reappraisal, and more expressive suppression, compared to parents scoring below cut-off.

**Table 2:** Participants’ reported levels of psychological distress, parenting concerns, self-efficacy, and emotion regulation (*n* = 406).

Measures	*n*	%	Mean	SD
**DASS**				
Depression				
No, below cut-off	266	65		
Yes, above cut-off	140	35		
Depression level				
Normal	214	53		
Mild	52	13		
Moderate	77	19		
Severe	26	6		
Extremely severe	37	9		
Anxiety				
No, below cut-off	279	69		
Yes, above cut-off	127	31		
Anxiety level				
Normal	244	60		
Mild	35	9		
Moderate	66	16		
Severe	21	5		
Extremely severe	40	10		
Stress				
No, below cut-off	298	73		
Yes, above cut-off	108	27		
Stress level				
Normal	241	59		
Mild	57	14		
Moderate	50	12		
Severe	47	12		
Extremely severe	11	3		
Comorbidity				
Depression and anxiety, yes	86	21		
Depression and stress, yes	81	20		
Anxiety and stress, yes	77	19		
Depression, anxiety and stress, yes	64	16		
**Parenting concerns** (PCQ) (mean)				
Practical impact on children			2.50	0.98
Emotional impact on children			2.18	0.91
Concerns about co-parent*			2.12	0.98
Total score			2.31	0.79
**Self-efficacy** (GSE) **score**			28.36	5.09
**Emotion regulation** (ERQ)				
Cognitive reappraisal score			4.54	1.10
Expressive suppression score			3.03	0.98

* Only participants with a partner were analysed, *n* = 336.

**Table 3:** Levels of parenting concerns, self-efficacy and emotion regulation in relation to presence or absence of depression, anxiety, and stress (*n* = 406).

Variables	*Depression*					*Anxiety*					*Stress*					*Depression, anxiety, and stress*				
	No		Yes			No		Yes			No		Yes			No		Yes		
	*n* = 266 (65%)		*n* = 140 (35%)			*n* = 279 (69%)		*n* = 127 (31%)			*n* = 298 (73%)		*n* = 108 (27%)			*n* = 342 (84%)		*n* = 64 (16%)		
	Mean	SD	Mean	SD	Mean diff.^a^ (Cohen’s *d*)^b^	Mean	SD	Mean	SD	Mean diff.^a^ (Cohen’s *d*)^b^	Mean	SD	Mean	SD	Mean diff.^a^ (Cohen’s *d*)^b^	Mean	SD	Mean	SD	Mean diff.^a^ (Cohen’s *d*)^b^
**Parenting concerns *(PCQ)***	2.08	0.68	2.73	0.81	0.65** (0.89)	2.13	0.71	2.7	0.79	0.58** (0.79)	2.13	0.69	2.81	0.82	0.69** (0.94)	2.20	0.73	2.93	0.80	0.73** (0.97)
**Self-efficacy *(GSE)***	29.29	4.72	26.59	5.30	-2.7** (-0.55)	29.18	4.75	26.57	5.36	-2.6** (-0.53)	29.10	4.58	26.31	5.83	-2.8** (-0.57)	28.92	4.73	25.36	5.85	-3.56** (-0.72)
**Cognitive reappraisal *(ERQ)***	4.72	1.01	4.21	1.18	-0.51** (-0.47)	4.64	1.08	4.33	1.10	-0.31* (-0.29)	4.67	1.02	4.19	1.23	-0.48** (-0.44)	4.61	1.06	4.17	1.22	-0.44* (-0.41)
**Expressive suppression *(ERQ)***	2.83	1.15	3.39	1.24	0.56** (0.47)	2.92	1.19	3.24	1.21	0.32* (0.26)	2.94	1.15	3.26	1.34	0.31* (0.26)	2.94	1.19	3.48	1.25	0.53* (0.45)

* *p* < 0.05.

** *p* < 0.001.

a Difference in means were computed using independent samples *t***-**tests.

b Cohen’s *d* effect size = 0.20 small, 0.50 medium, and 0.80 large.

### Variables associated with depression, anxiety and stress

Results of the unadjusted and adjusted logistic regression analyses are shown in Table 4.

**Table 4:** Logistic regression models of variables associated with depression, anxiety, and stress (*n* = 406).

Variables	Cat.	*Depression*		*Anxiety*		*Stress*		*Depression, anxiety, and stress*	
		OR (95% CI)	aOR (95% CI)^a^	OR (95% CI)	aOR (95% CI)^b^	OR (95% CI)	aOR (95% CI)^c^	OR (95% CI)	aOR (95% CI)^d^
***Age***	<40 40–50 >50	Ref. 0.93 (0.55–1.59) 0.83 (0.43–1.59)	Ref. 1.15 (0.54–2.451) 1.45 (0.52–4.065)	Ref. 0.76 (0.45–1.29) 0.55 (0.28–1.08)	Ref. 0.57 (0.27–1.2) 0.43 (0.16–1.20)	Ref. 0.63 (0.36–1.09) 0.54 (0.27–1.08)	Ref. 0.63 (0.29–1.35) 0.78 (0.27–2.29)	Ref. 0.63 (0.33–1.19) 0.48 (0.20–1.11)	Ref. 0.56 (0.23–1.38) 0.54 (0.14–2.06)
***Parent***	Mother Father	Ref. 0.95 (0.33–2.25)	Ref. 0.80 (0.23–2.81)	Ref. 0.62 (0.19–1.90)	Ref. 0.78 (0.21–2.81)	Ref. 0.33 (0.08–1.47)	Ref. 0.45 (0.09–2.39)	Ref. 0.30 (0.40–2.32)	Ref. 0.43 (0.04–4.08)
***Education***	Post-sec. Secondary	Ref. 1.41 (0.83–2.41)	Ref. 0.99 (0.51–1.90)	Ref. 1.82 (1.07–3.10)*	Ref. 1.52 (0.82–2.82)	Ref. 1.18 (0.66–2.11)	Ref. 1.09 (0.55–2.17)	Ref. 1.67 (0.87–3.19)	Ref. 1.38 (0.62–3.05)
***Partner***	Yes No	Ref. 1.15 (0.67–1.96)	Ref. 1.31 (0.68–2.50)	Ref. 0.93 (0.53–1.62)	Ref. 0.94 (0.49–1.78)	Ref. 0.79 (0.43–1.44)	Ref. 0.74 (0.37–1.50)	Ref. 0.87 (0.42–1.80)	Ref. 0.88 (0.38–2.06)
***No. of children***		1.04 (0.84–1.28)	0.99 (0.77–1.28)	1.12 (0.91–1.39)	1.09 (0.85–1.40)	1.02 (0.81–1.28)	0.96 (0.74–1.26)	1.10 (0.85–1.43)	1.11 (0.81–1.52)
***Age of youngest child***		0.99 (0.95–1.03)	0.97 (0.912–1.04)	0.99 (0.95–1.04)	1.02 (0.96–1.09)	0.97 (0.92–1.01)	0.97 (0.91–1.41)	0.98 (0.94–1.03)	1.01 (0.93–1.09)
***Time since diagnosis***	3+ <1 1–2	Ref. 1.12 (0.65–1.93) 1.06 (0.62–1.81)	Ref. 0.96 (0.50–1.88) 0.88 (0.45–1.70)	Ref. 0.89 (0.52–1.54) 0.80 (0.47–1.34)	Ref. 0.74 (0.40–1.39) 0.60 (0.32–1.14)	Ref. 0.84 (0.48–1.48) 0.68 (0.39–1.19)	Ref. 0.72 (0.37–1.42) 0.49 (0.25–0.97)*	Ref. 1.29 (0.63–2.60) 1.02 (0.50–2.09)	Ref. 1.38 (0.58–3.28) 0.82 (0.35–1.96)
***Psychological distress before cancer diagnosis***	No Yes	Ref. 2.41 (1.58–3.68)**	Ref. 1.63 (0.97–2.72)	Ref. 1.77 (1.15–2.71)*	Ref. 1.12 (0.68–1.85)	Ref. 2.41 (1.54–3.78)**	Ref. 1.48 (0.86–2.52)	Ref. 2.27 (1.32–3.89)*	Ref. 1.29 (0.67–2.46)
***Incurable cancer***	No Yes	Ref. 1.67 (0.99–2.81)	Ref. 1.54 (0.81–2.94)	Ref. 1.04 (0.60–1.79)	Ref. 0.76 (0.40–1.44)	Ref. 0.62 (0.33–1.16)	Ref. 0.38 (0.18–0.80)*	Ref. 0.52 (0.23–1.20)	Ref. 0.32 (0.12–0.83)*
***Self-rated health***	Good Poor	Ref. 4.90 (3.15–7.61)**	Ref. 3.28 (1.98–5.41)**	Ref. 3.14 (2.03–4.86)**	Ref. 1.97 (1.21–3.22)*	Ref. 2.61 (1.66–4.10)**	Ref. 1.42 (0.84–2.43)	Ref. 4.89 (2.67–8.97)**	Ref. 2.71 (1.37–5.34)*
***Parenting concerns (PCQ)***		3.11 (2.30–4.22)**	2.33 (1.64–3.31)**	2.68 (1.99–3.59)**	2.30 (1.64–3.20)**	3.21 (2.34–4.42)**	3.21 (2.20–4.69)**	3.28 (2.27–4.73)**	3.01 (1.93–4.70)**
***Self–efficacy (GSE)***		0.90 (0.86–0.94)**	0.97 (0.92–1.02)	0.90 (0.86–0.94)**	0.94 (0.89–0.99)*	0.90 (0.86–0.94)**	0.95 (0.90–1.01)	0.89 (0.83–0.92)**	0.93 (0.86–0.99)*
***Cognitive reappraisal (ERQ)***		0.65 (0.53–0.79)**	0.76 (0.59–0.98)*	0.77 (0.64–0.94)*	1.01 (0.80–1.23)	0.67 (0.55–0.83)**	0.84 (0.65–1.09)	0.70 (0.55–0.89)*	0.98 (0.72–1.34)
***Expressive suppression (ERQ)***		1.48 (1.24–1.77)**	1.46 (1.18–1.80)**	1.24 (1.04–1.48)*	1.10 (0.91–1.35)	1.24 (1.03–1.49)*	1.16 (0.94–1.43)	1.44 (1.15–1.79)**	1.30 (1.00–1.69)*

* *p* < 0.05.

** *p* < 0.001.

a Nagelkerke *R*^2^ = 0.37, correct classification percentage = 78.6%.

b Nagelkerke *R*^2^ = 0.25, correct classification percentage = 73.4%.

c Nagelkerke *R*^2^ = 0.32, correct classification percentage = 77.3%.

d Nagelkerke *R*^2^ = 0.34, correct classification percentage = 84.7%.

#### Depression

In unadjusted analyses, psychological distress before cancer diagnosis, poor self-rated health, greater parenting concerns, and higher expressive suppression were associated with higher odds of depression, whereas higher self-efficacy and cognitive reappraisal were associated with lower odds.

In the adjusted model, poor self-rated health was associated with three times greater odds of depression (OR = 3.28, 95% CI: 1.98–5.41, *p* < 0.001). The odds of depression more than doubled with each unit increase in parenting concerns (OR = 2.33, 95% CI: 1.64–3.31, *p* < 0.001). Greater use of cognitive reappraisal was associated with lower odds of depression (OR = 0.76, 95% CI: 0.59–0.98, *p* < 0.05) whereas using more expressive suppression was associated with higher odds of depression (OR = 1.46, 95% CI: 1.18–1.80, *p* < 0.001).

The Hosmer and Lemeshow test showed good goodness of fit for the adjusted model (HL: χ² = 11.346, df = 8, *p* = 0.183).

#### Anxiety

Lower level of education, psychological distress before cancer, poor self-rated health, greater parenting concerns, and higher expressive suppression increased the odds for anxiety in unadjusted analyses, while higher use of cognitive reappraisal decreased the odds.

In adjusted analyses, poor self-rated health was associated with a nearly two-fold greater odds of anxiety (OR = 1.97, 95% CI: 1.21–3.22, *p* < 0.05). For each unit increase in parenting concerns, the odds of anxiety more than doubled (OR = 2.30, 95% CI: 1.64–3.20, *p* < 0.001), whereas for each unit increase in self-efficacy, the odds of anxiety decreased by 6% (OR = 0.94, 95% CI: 0.89–0.99, *p* < 0.05). The adjusted model had a good fit (HL: χ² = 3.77, df = 8, *p* = 0.877).

#### Stress

In unadjusted analyses, psychological distress before cancer diagnosis, poor self-rated health, higher parenting concerns, and higher use of expressive suppression increased the odds for stress. Higher self-efficacy and cognitive reappraisal lowered the odds for stress.

In the adjusted model, parenting concerns were associated with stress (OR = 3.21, 95% CI: 2.20–4.69, *p* < 0.001), tripling the odds for stress with each unit increase in parenting concerns. Time since diagnosis (1–2 years vs. 3+ years) (OR = 0.49, 95% CI: 0.25–0.97, *p* < 0.05) and incurable cancer (OR = 0.38, 95% CI: 0.18–0.80, *p* < 0.05) decreased the odds for stress by 51 and 62%, respectively. The adjusted model showed a good fit (HL: χ² = 5.513, df = 8, *p* = 0.702).

#### Depression, anxiety, and stress

The odds of reporting depression, anxiety, and stress were higher in unadjusted analyses if parents experienced psychological distress before cancer diagnosis, poor self-rated health, higher parenting concerns, lower self-efficacy, lower use of cognitive reappraisal, and higher use of expressive suppression.

In the adjusted model, poor self-rated health more than doubled the odds for comorbidity (OR = 2.71, 95% CI: 1.37–5.34, *p* < 0.05), parenting concerns tripled the odds (OR = 3.01, 95% CI: 1.93–4.70, *p* < 0.001), and each unit increase of expressive suppression increased the odds by 30% (OR = 1.30, 95% CI: 1.00–1.69, *p* < 0.05). Incurable cancer (OR = 0.32, 95% CI: 0.12–0.83, *p* < 0.05) and self-efficacy (OR = 0.93, 95% CI: 0.86–0.99, *p* < 0.05) decreased the odds for comorbidity. The adjusted model showed a good fit (HL: χ² = 7.723, df = 8, *p* = 0.461).

## Discussion

This study aimed to explore psychological distress (depression, anxiety, and stress) in parents with cancer and associations with parenting concerns, self-efficacy, and emotion regulation. One third of parents with cancer in this study scored above cut-off for depression, anxiety, or stress, which is similar [32] or in the lower range compared to other studies of adults with cancer [2, 3] and parents with advanced cancer [7], but higher than a study of mothers with cancer [33]. One reason for a lower level of psychological distress might be that in other studies different measures have been used, and often shortly after diagnosis when the risk of psychological distress is higher, while in our sample parents had received their diagnosis up to 5 years ago.

Parents reported mild to moderate parenting concerns, largely in line with previous findings [28, 34, 35], although in some studies concerns were lower [13] or higher [36]. The levels of parenting concerns found are not reflective of qualitative findings describing parenting concerns as a major stressor for adult cancer patients [10–12]. This may indicate that other factors play a part, for example that the positive aspects of having children may balance out some parenting concerns, or that the PCQ does not fully capture all aspects of parenting concerns. Nevertheless, greater parenting concerns were associated with greater odds of depression, anxiety, and stress, while adjusting for a range of other variables including age, gender, education, civil status, number of children, psychological distress before the cancer diagnosis, and self-rated health.

Parenting concerns doubled the odds for depression, in line with previous research [18]. Depression has also been shown to be the strongest predictor of parenting stress [23], highlighting the reciprocal relationship between parenting and psychological distress. In another study, the relationship between parenting concerns and depression was not significant when adjusting for parents’ levels of functioning (Eastern Cooperative Oncology Group (ECOG) performance status) [7]. In the present study, we did not adjust for ECOG status but for self-rated health, and parenting concerns remained significant. Self-rated health was also associated with depression, which was not surprising, since lower self-rated health has been seen as a predictor for depression in previous research [37].

Higher cognitive reappraisal was associated with lower odds for depression and higher expressive suppression increased the odds, substantiating the association between psychological distress and emotion regulation [21], where less use of cognitive reappraisal has been a predictor of maternal depression in cancer patients [22]. Receiving a cancer diagnosis is a major life event that leads to changes for the entire family. To be able to regulate one’s emotions in an adaptive way to cope with the disease and its consequences is probably important for reducing the risk of depression.

Neither expressive suppression nor cognitive reappraisal were associated with anxiety, but higher parenting concerns and poor self-rated health were associated with greater odds of anxiety, while higher self-efficacy was a protective factor. Parenting concerns doubled the odds for anxiety, which is in line with a study of parents with advanced cancer where parenting concerns increased the odds for anxiety by 50% [7]. Low self-efficacy has previously been linked to anxiety, but also to depression and psychological distress in general [19, 33], as well as parenting concerns [38]. Interestingly, we only observed an association between self-efficacy and anxiety, while adjusting for other factors. This may be related to the self-efficacy levels reported being equivalent to an adult population without cancer [39], or that other factors were stronger predictors. The use of a parenting efficacy scale instead might have given different results, as seen in other studies where parenting efficacy beliefs decreased after a cancer diagnosis [19]. Further, the DASS-21 measures physical symptoms of anxiety rather than worry, which indicates that another instrument may be more relevant in cancer patients who often worry about their children, and their own potential decline in health and possible death [7].

Greater parenting concerns, shorter time since diagnosis, and curable cancer were associated with stress. Parents often experience stress, in particular parenting stress [22], which might explain why parenting concerns were so strongly associated with stress, more so than depression and anxiety. The burden associated with being both a parent and a patient has been described previously, and parents lack resources for this, which can result in more stress [12, 24]. Previous studies have shown incurable cancer to be associated with higher psychological distress [8, 11], whereas in this study the odds of stress were lower among those who reported their cancer to be incurable. One possible explanation for this may be related to time since diagnosis. With time, the initial shock of receiving the diagnosis has usually passed and the family may have found ways to adapt to, and cope with, the cancer and its effects. Another explanation might be the variety of experiences when having incurable cancer depending on disease progression, for example receiving end-of-life treatment or not [7, 28].

### Strengths and limitations

A major strength of this study is the fact that it included many variables to investigate psychological distress among parents, which no previous study has done in this population. Another strength was a large sample of parents diagnosed with cancer within 5 years. Previous studies have indicated that time since diagnosis affects well-being and we were able to explore the association with time since diagnosis cross-sectionally. However, as in many studies, one limitation was the homogenous sample where the majority were mothers with breast cancer. A sensitivity analysis was performed, excluding fathers with cancer from the logistic regression models, and results remained the same (data not shown). Thus, we opted to keep the full study sample in the analysis. In addition, we targeted an age range (25–60 years) that would encompass parents more likely to have dependent children, but excluded parents under 25 and over 60. This is another limitation, since younger or older parents may have different challenges; the sample therefore limits the generalisability of the results. With a convenience sample, the representativeness of the sample is unknown and could include an overrepresentation of either parents with particular high concerns or distress or the opposite, that parents with extreme concerns or distress did not participate, impacting the external validity of the study. In addition, this study only includes the sick parent and as such, any potential dyadic effects on parenting concerns and psychological distress could not be addressed. Where parenting is shared, studies have shown the influence of co-parents on psychological well-being and parenting concerns [24]. Social support and peer support outside the family has also been shown to be important for psychological well-being [11], which this study did not control for.

Further, the cross-sectional design of the study precludes any conclusions regarding causality, which must be taken into consideration when interpreting the results. Nevertheless, the results still shed light on what may be important factors to address in psychological interventions for parents with cancer, something that has been called for [10, 21]. A limited number of interventions to alleviate psychological distress in parents with cancer have been formally evaluated, where a couple have been shown to be effective (e.g., the Enhancing Connections and Wonders & Worries) [36, 40]. These interventions are predominantly characterised by patient counselling sessions with psychoeducational content in contrast to more experiential methods. In this study, we were interested in studying associations between psychological distress and modifiable factors that can be targeted in psychological treatment (i.e., self-efficacy beliefs, emotion regulation capacity, and parenting concerns). Although previous studies have shed light on some factors relevant for psychological distress among parents with cancer when addressed as an outcome [11, 18, 21, 22], we considered adding multiple of these factors; parenting concerns, self-efficacy and emotion regulation to the analyses as an important contribution that can result in reducing psychological distress in this population in the future by providing parents with more effective psychological treatments.

## Conclusion

In conclusion, psychological distress was associated with elevated parenting concerns, lower self-efficacy, and maladaptive emotion regulation in parents with cancer who have dependent children. These findings underscore the multifaceted nature of parents’ well-being in relation to parenthood and cancer, emphasising the importance of interventions targeting relevant psychological factors (i.e. parenting concerns, self-efficacy, and emotion regulation) to enhance psychological well-being in this population.

## Data Availability

The raw data are not publicly available due to ethical restrictions since they contain sensitive data. The data that support the findings of this study are available from the corresponding author, upon reasonable request and with necessary ethics approval.
